# Acetylshikonin induces autophagy‐dependent apoptosis through the key LKB1‐AMPK and PI3K/Akt‐regulated mTOR signalling pathways in HL‐60 cells

**DOI:** 10.1111/jcmm.17202

**Published:** 2022-02-02

**Authors:** Meng‐Di Wu, Yuan‐Ying Zhang, Shu‐Ying Yi, Bei‐Bei Sun, Jing Lan, Han‐Ming Jiang, Gang‐Ping Hao

**Affiliations:** ^1^ School of Basic Medical Sciences Shandong First Medical University &Shandong Academy of Medical Sciences Jinan China

**Keywords:** acetylshikonin (ASK), apoptosis, autophagy, HL‐60 cells, LKB1/AMPK, PI3K/Akt/mTOR

## Abstract

Acetylshikonin (ASK) is a natural naphthoquinone derivative of traditional Chinese medicine *Lithospermum erythrorhyzon*. It has been reported that ASK has bactericidal, anti‐inflammatory and antitumour effects. However, whether ASK induces apoptosis and autophagy in acute myeloid leukaemia (AML) cells and the underlying mechanism are still unclear. Here, we explored the roles of apoptosis and autophagy in ASK‐induced cell death and the potential molecular mechanisms in human AML HL‐60 cells. The results demonstrated that ASK remarkably inhibited the cell proliferation, viability and induced apoptosis in HL‐60 cells through the mitochondrial pathway, and ASK promoted cell cycle arrest in the S‐phase. In addition, the increased formation of autophagosomes, the turnover from light chain 3B (LC3B) I to LC3B II and decrease of P62 suggested the induction of autophagy by ASK. Furthermore, ASK significantly decreased PI3K, phospho‐Akt and p‐p70S6K expression, while enhanced phospho‐AMP‐activated protein kinase (AMPK) and phospho‐liver kinase B1(LKB1) expression. The suppression of ASK‐induced the conversion from LC3B I to LC3B II caused by the application of inhibitors of AMPK (compound C) demonstrated that ASK‐induced autophagy depends on the LKB1/AMPK pathway. These data suggested that the autophagy induced by ASK were dependent on the activation of LKB1/AMPK signalling and suppression of PI3K/Akt/mTOR pathways. The cleavage of the apoptosis‐related markers caspase‐3 and caspase‐9 and the activity of caspase‐3 induced by ASK were markedly reduced by inhibitor of AMPK (compound C), an autophagy inhibitor 3‐methyladenine (3‐MA) and another autophagy inhibitor chloroquine (CQ). Taken together, our data reveal that ASK‐induced HL‐60 cell apoptosis is dependent on the activation of autophagy via the LKB1/AMPK and PI3K/Akt‐regulated mTOR signalling pathways.

## 1 INTRODUCTION

Acute myelogenous leukaemia (AML) is a neoplasm of immature myeloid cells, and it is the second most common blood cancer in humans.^1^ AML is caused by the overproduction of immature myeloid cells in the bone marrow, which can lead to bone marrow failure and organ infiltration. AML has an aggressive clinical course, and the overall five‐year survival rate is approximately 25%.^1^ Over the past decades, the refinement of supportive therapies, such as chemotherapy and haematopoietic stem cell therapy, has improved the prognosis of AML patients. However, more than 90% of older patients and 50% of younger patients still die from the disease.^2^ The treatment outcome of AML appears to be worse with increasing age.^3^ Older AML patients often cannot tolerate treatments such as chemotherapy and haematopoietic stem cell therapy. Resistance to chemotherapeutic drugs, as regulated by the expression of multidrug resistance (MDR) genes, is also more frequently observed in elderly AML patients.^4^ The prognosis of AML patients is poor, and most die within a year of diagnosis.^5^ Therefore, it is very important to find highly effective natural drugs with less side effects for treating acute myeloid leukaemia. The combination of chemotherapeutics and traditional Chinese herbs may be an effective treatment option for patients with AML.

Autophagy, a highly conserved pathway in eukaryotic cells that is mediated by lysosomes and degrades cytosolic components, is an important process that can degrade and recycle cellular components. It is triggered in response to diverse stress and/or extrinsic stimuli.^6^ Autophagy integrates several signalling pathways to regulate multiple cellular functions, such as cell metabolism, growth, proliferation and survival.^6^ Mammalian target of rapamycin (mTOR) is a major negative regulator of mammalian autophagy as controlled by growth receptors, nutrient depletion, hypoxia, oxidative stress or low energy.^7^ Inhibition of mTOR will lead to the autophagy induction,^8^ and mTOR repression could be activated by the LKB1/AMPK signalling and be inhibited by phosphatidylinositol 3 kinase (PI3K)/protein kinase B (Akt) signalling.^8^

A number of studies suggest that LKB1/AMPK and PI3K/Akt signalling pathway serves a key role in the occurrence and development of tumours.^9^, ^10^ The LKB1/AMPK signalling pathway is regarded as tumour suppressor axis in recent years.^11^ In acute myeloid leukaemia, LKB1/AMPK signalling pathway has tumour suppressive activity by inhibiting mTOR‐dependent oncogenic mRNA translation.^12^ The activation of AMPK can promote autophagy, and cyclinD1 inhibits the autophagy induced by oncogenes by regulating the AMPK‐LKB1 signalling pathway.^13^ The results of mice transplanted with a human AML cell line suggest that AMPK agonists may become a new perspective for AML therapy.^12^ Activation of PI3K/Akt/mTOR pathway seems to be important for the development of leukaemia, including acute myeloid leukaemia (AML).^9^ The activation of PI3K/Akt/mTOR pathway in cancers leads to rapid proliferation, apoptosis escape and chemoresistance of tumour cells.^14^ PI3K/Akt /mTOR pathway was demonstrated to be as the modulators of autophagy. Therefore, targeting PI3K/Akt/mTOR‐mediated autophagy has emerged as a possible tumour therapeutic strategy. Tanshinone, extracted from *Salvia miltiorrhiza*, can induce autophagy and apoptosis by inhibiting the PI3K/Akt/mTOR pathway in ovarian cancer cells.^15^

The root of traditional Chinese herbs *Lithospermum erythrorhizon*, named Zicao, has been widely used in the treatment of burns and scalds, allergic purpura, measles, rashes and yellow spots.^16^ Shikonin and acetylshikonin are the main active components of *Lithospermum erythrorhizon*. Shikonin and its analogues exhibit strong anticancer and anti‐inflammatory properties, but with potential adverse effects.^16^ A derivative of shikonin, acetylshikonin (ASK), is less toxic to normal cells than shikonin, so it could be used as a potential anticancer drug.^17^, ^18^ It has been reported that ASK has antitumour effects and can induce apoptosis in squamous cell carcinoma, melanoma cells and colorectal cancer cells through inhibition of the PI3K/Akt pathway and induction of Foxo nuclear translocation.^19^, ^20^, ^21^, ^22^ ASK is also responsible for ameliorating autophagy in nonalcoholic steatohepatitis via AMPK/mTOR pathway.^23^ However, no studies have investigated the effects of ASK on acute myelogenous leukaemia (AML), and the apoptotic and autophagy mechanism of acetylshikonin against AML cells is still not thoroughly investigated. The goals of this study were to improve our understanding of the effects of ASK on HL‐60 cells derived from a patient with AML FAB‐M2,^24^ to study the mechanisms involved and to evaluate the therapeutic potential of ASK in treating acute myelogenous leukaemia (AML).

## 2 MATERIALS AND METHODS

### 2.1 Cell lines and reagents

Acetylshikonin was purchased from Shanghai ShiFeng Chemical Co. (Shanghai, China), and its chemical had a purity of >98%. All cell lines used in this study were obtained from the Cell Resource Center of the Shanghai Institutes for Biological Sciences of the Chinese Academy of Sciences. DMSO was purchased from Sigma (No. D4540). DAPI and PI were obtained from Solarbio (Beijing, China). EdU Cell Proliferation Detection Kit was purchased from RiboBio Co. (Guangzhou, China). Annexin V‐FITC was purchased from BD Biosciences (San Jose, CA). Caspase‐Glo^®^ 3/7 Assay was purchased from Promega Corporation (Madison, USA). Compound C (sc‐200,689) was purchased from Santa Cruz Biotechnology Inc (Santa Cruz, CA, USA). 3‐methyladenine (3‐MA) and Chloroquine (CQ) were obtained from Sigma‐Aldrich (St. Louis, MO, USA). Anti‐LC3B antibody‐Autophagosome Marker (ab48394) was purchased from Abcam (Cambridge, UK). Antibodies against poly (adenosine diphosphate [ADP]‐ribose) polymerase (PARP) (Asp214) (No. 9532), caspase‐3 (Asp175) (5A1E) (No. 9662), caspase‐9 (No. 9502), Bax (No. 2772), Bcl‐2(124) (No. 15071), CDK2(78B2) (No. 2546), AMPKα (No. 2532), p‐AMPKα (Thr172) (40H9) (No. 2535), p‐LKB1 (Ser428) (C67A3) (No. 3482), p70S6 Kinase (49D7) (No. 2708), P21 (12D1) (No. 2947), p‐AKT (Ser473) (D9E) (No. 4060), AKT (C67E7) (No. 4691) and p‐Raptor (24C12) (No.2280S) were purchased from Cell Signaling Technology (Beverly, MA). Antibodies against cytochrome c (A‐8) (No. SC‐13156), apoptosis‐inducing factor (AIF, E‐1) (No. SC‐13116), P62 (SQSTM1) (No. SC‐28359), LKB1 (No. SC‐32245), cyclin E (HE12) (No. SC‐247), PI3K p85α (B‐9) (No. SC‐1637), p‐P70S6 (A‐6) (No. SC‐8416) and Raptor (10E10) (No. SC‐81537) were purchased from Santa Cruz Biotechnology (Santa Cruz, CA). Anti‐LC3B (ab51520), ATG5 (ab108327), ATG7 (ab52472), Beclin‐1 (2A4) (ab114071) and cyclin A2 (ab181591) were purchased from Abcam (Cambridge, UK). The dye mix for the EB/AO staining was 100 μg/mL acridine orange and 100 μg/mL ethidium bromide in PBS.

### 2.2 Cell culture

HL‐60 and K562 cells were grown in Iscove's modified Dulbecco's medium (IMDM, HyClone) supplemented with 10% foetal bovine serum (FBS, Gibco) and 100 IU/mL penicillin and 100 μg/mL streptomycin at 37°C and 5% CO_2_. THP‐1 cells were maintained in RPMI‐1640 medium (sigma) supplemented with 10% foetal bovine serum (FBS, Gibco). The cells were prepared for subculture every 24–48 h. The log phase cells with >98% activity were employed for subsequent experiments.

### 2.3 Assessment of cell proliferation inhibition by XTT assay

HL‐60, K562 and THP‐1 cells (1.6 × 10^5^ cells/mL) were grown in a 96‐well plate and then exposed to 0.3125–5 µM ASK. The same amount of DMEM or RPMI‐1640 was employed as the blank control. A minimum of 6 replicates was made at each concentration, and the experiments were conducted in a total volume of 100 µL/well. The cells treated without ASK were employed as the control group. After incubation for 24–48 h, XTT (50 μL/well) was added and incubated again for 4 h. The absorbance (A) values were detected using a BioRad M450 microplate reader at 490 nm. All experiments were repeated 3 times, and the cellular proliferation inhibition rate (CPIR) was determined as follows: CPIR = (1 ‐ mean A490 value of the treatment group/mean A490 value of the control group) × 100%. GraphPad Prism was used to calculate the IC_50_ values.

### 2.4 Assessment of cell proliferation by EdU assay

HL‐60, K562 and THP‐1 cells (2.0 × 10^5^ cells/mL) were exposed to 0.3125–5 µM ASK for 24 h. The cells treated without ASK were employed as the control group. Cell proliferation was measured using EdU kits (RIBOBIO, Guangzhou, China) according to the manufacturer's instructions to analyse the incorporation of EdU during DNA synthesis. Cells were collected by centrifugation and incubated with 50 μM EdU for 2 h. The supernatant was discarded after the cells were collected by centrifugation. Wash cells with PBS for one time, and then fix cells with methanol for 30 min. The cell membranes were lysed with 0.5% Triton X‐100 in a shaking table. Then, the 1xApollo^®^ dyeing reaction solution was added to the cells and cultured for 30 min without light. The cell nucleus was stained with Hoechst 33322 for 30 min. The proportion of cells incorporated EdU was determined by fluorescence microscopy (Invitrogen™ EVOS FL Auto 2, Thermo Fisher Scientific, America). Assays were performed in triplicate.

### 2.5 Determination of cell viability by Trypan blue assay

Cell viability was evaluated using the trypan blue exclusion test. HL‐60 cells (2.0 × 10^5^ cells/mL) were exposed to 0.3125–5 µM ASK in a 6‐well plate. The cells treated without ASK were employed as the control group. After incubation for 48 h, the cells were trypsinized, resuspended in medium and stained with trypan blue dye (0.4%) for 5 min. The blue‐stained (non‐viable) and unstained (viable) cells were measured using a haemocytometer. The cellular death rate (CDR) was calculated using the following equation: CDR = (1 ‐ mean number of living cells in the experimental group/mean number of living cells in the control group) × 100%. All experiments were repeated 3 times.

### 2.6 Cell cycle analysis

HL‐60 cells (1.5 × 10^5^ cells/mL) were exposed to 0.3125–5 µM ASK for 24 h. After harvesting, rinsing with PBS and fixing in 70% ethanol overnight, the cells were centrifuged and rinsed again with PBS. Then, the cells were incubated with propidium iodide (PI, 50 μg/mL) and RNase (2.5 μg/mL in PBS) at room temperature (RT) for 30 min. The DNA content was analysed using a flow cytometer at 488 nm. All experiments were repeated 3 times.

### 2.7 Apoptosis evaluation

HL‐60 cells (1.5 × 10^5^ cells/mL) were grown in 6‐well plates and then exposed to 0.3125–5 µM ASK for 24 h. Apoptotic cells were assessed by annexin V/PI double staining using a flow cytometer. After resuspension in 1× binding buffer (100 µL), the cells were incubated with annexin V (5 µL) and PI (5 µL) at RT for 15 min in the dark. Subsequently, 500 µL of the above‐mentioned buffer was added, and flow cytometric analysis was conducted with BD FACSCanto II. All experiments were repeated three times.

### 2.8 Examination of apoptotic morphology by ethidium bromide (EB)/acridine orange (AO) staining

HL‐60 cells (2.0 × 10^5^ cells/mL) were grown in a 6‐well plate and then exposed to 0.3125–5 µM ASK for 24 h. After harvesting and rinsing with PBS, 50 µL cell suspension (0.5–2.0 million cells/mL) was stained with 1 µL EB/AO solution (100 μg/mL). Then, 25 µL stained cell suspension was transferred onto a microscopic slide, covered with a glass coverslip and observed under a fluorescence microscope. A minimum of 100 stained cells in each sample were counted. All experiments were repeated three times.

### 2.9 Caspase‐3 activity assay

Caspase‐3 activities were analysed with Caspase‐Glo 3/7 assay kit (Promega, G8091) in accordance with the Caspase‐Glo 3/7 assay protocol. Exponentially growing HL‐60 (1.6 × 10^5^ cells/mL) cells were grown in a 96‐well plate and then exposed to 0.3125–5 µM ASK. The same amount of DMEM was employed as the blank control. A minimum of six replicates was made at each concentration, and the experiments were conducted in a total volume of 100 µL/well. The cells treated without ASK were employed as the control group. After incubation for 24 h, 100 μL Caspase‐Glo 3/7 assay reagent was added and mixed gentle at 300–500 rpm for 30 s using a plate shaker. The samples were incubated at RT for 1 h. Finally, luminescent measurement was performed on a plate‐reading luminometer by following the manufacturer's instructions (GloMax Navigator, Promega, Madison, USA).

### 2.10 Immunofluorescence

The expression of microtubule‐associated protein 1 light chain 3 beta (LC3B), a general marker for autophagic membranes,^25^ was analysed by immunofluorescence and Western blotting. HL‐60 cells were exposed to 0, 0.625 and 1.25 μM ASK for 24 h. After harvesting and rinsing with PBS, the cells were fixed with ice‐cold methanol (100%) for 20 min and permeabilized with Triton X‐100 (0.1%) for 10 min. After blocking with BSA (3%) at RT for 1 h, the cells were incubated with a 1:300 dilution of primary anti‐LC3B antibody at RT for 2 h and then with CY3‐conjugated anti‐rabbit secondary antibody at RT for 1 h in the dark. The cells were washed repeatedly with PBS. After adding DAPI to counterstain the nucleus and incubating the cells with the dye at RT for 1 h, the cells were rinsed with PBS. Fluorescence was imaged with a fluorescence microscope (Invitrogen™ EVOS FL Auto 2, Thermo Fisher Scientific, America).

### 2.11 Transmission electron microscopy (TEM)

Transmission electron microscopy examination of ASK‐treated HL‐60 cells was conducted to determine autophagy‐induced cell death. HL‐60 cells were exposed to 0, 0.625 and 1.25 µM ASK for 24 h. After harvesting and rinsing with PBS, the cells were fixed with glutaraldehyde (2%) in 0.1 M PBS and then postfixed with osmium tetroxide (1%). The cells were then incubated with uranyl acetate (1%), dehydrated with a graded series of ethanol and infiltrated with LX112 solution. After embedding in epoxy resin, the samples were sectioned and stained with uranyl acetate (2%) and lead citrate. Lastly, the sections were observed using a transmission electron microscope (HT‐7700, Hitachi, Japan).

### 2.12 Quantitative real‐time PCR (RT‐qPCR)

HL‐60 cells (2.0 × 10^5^ cells/mL) were exposed to 0.3125–5 µM ASK for 24 h and 1.25 µM ASK for 6–24 h. Total RNA was extracted using TRIzol^®^ (Invitrogen), and the concentration of all RNA samples was detected. Afterwards, cDNA was synthesized using a ReverTra Ace qPCR RT Kit (TOYOBO; Catalog number: TYB‐FSQ‐101). Next, qRT‐PCR was performed for detecting Akt and MAPKα1 mRNA expression after treated with ASK by using SYBR Select Master Mix (Thermo Fisher; Catalog number: 4472919). The primers used for RT‐qPCR were described in Table 1 to detect expression levels of Akt1, Akt2, Akt 3 and MAPKα1. The relative mRNA expression of MAPKα1 and Akt1‐3 was calculated via the 2^−ΔΔCt^ method and normalized to GAPDH.

**TABLE 1:** Sequences of primers used for Quantitative real‐time PCR analysis

Gene name	Forward primer (5′−3′)	Reverse primer (5′−3′)
Akt 1	GCAGCACGTGTACGAGAAGA	GGTGTCAGTCTCCGACGTG
Akt 2	AGTCCCCACTCAACAACTTCT	GAAGGTGCGCTCAATGACTG
Akt 3	TGCCTCTACAACCCATCATAAA	TCCACTTGCCTTCTCTCGAA
MAPKα1	GATTCGGAGCCTTGATGTGG	TCCTGAGACATATTCCATCACCA

### 2.13 Total protein extraction, isolation of cytosolic and mitochondrial fractions and Immunoblotting analysis

After harvesting and rinsing with PBS, the cells were subjected to protein extraction. Total proteins were extracted with PMSF‐containing RIPA lysis buffer (Solarbio, China). Cytosolic and mitochondrial proteins were extracted with Cytoplasmic and Mitochondrial Protein Extraction Kit (Sangon Biotech, C500051, Shanghai, China) according to the manufacturer's protocol, and protein concentration was assessed with the BCA method (Beyotime, P0012). The protein samples were then loaded and separated by SDS‐PAGE gels. After transferring onto a nitrocellulose membrane, primary antibody incubation was conducted at 4°C for 24 h, followed by secondary antibody incubation for 2 h. Lastly, enhanced chemiluminescence reagent was used to visualize the protein bands. GAPDH was employed as an internal control of total cell protein and cytosolic fraction; COXIV was used as mitochondrial fraction control.

### 2.14 Statistical analysis

All data were presented as mean ± standard deviation (SD). Statistical tests were performed with SPSS v16.0 software. Student's *t*‐test, one‐way ANOVA or repeated‐measures ANOVA was used to compare the differences among variables. The level of statistical significance was set at *p *< 0.05.

## 3 RESULTS

### 3.1 ASK attenuates the proliferation and viability of HL‐60 cells

**FIGURE 1:**
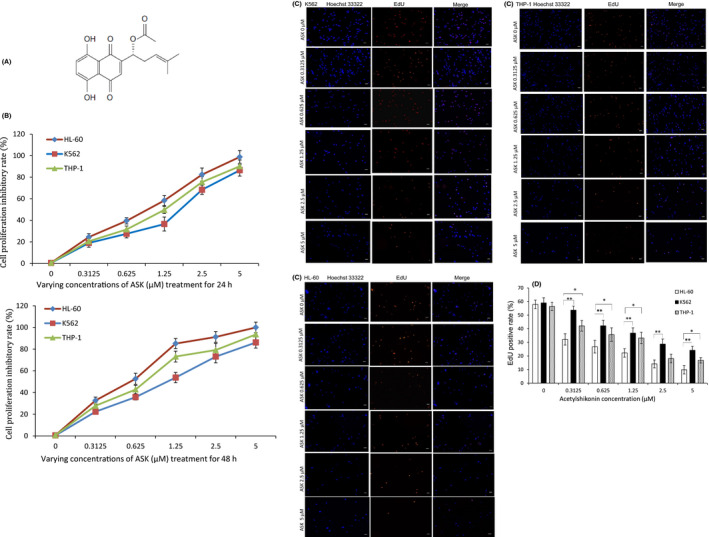
(A) Chemical structure of ASK. (B) A panel of leukaemia cell lines HL‐60, K562 and THP‐1 cells were treatment with 0–5 μM ASK for 24 and 48 h. Cell proliferation inhibiration was detected by XTT method. Mean ± SD, *n* = 6. (C, D) HL‐60, K562 and THP‐1 cells were treatment with 0–5 μM ASK for 24 h. Cell proliferation was detected by EdU assay. ×260; Scale bar = 20 μm. Mean ± SD, *n* = 3. *p < 0.05, **p < 0.01

This study aimed to investigate whether ASK can be employed as effective therapeutic option for AML. To address this, an XTT assay was used to assess the proliferation inhibition effect of ASK (Figure 1A) on a panel of leukaemia cell lines HL‐60, K562 and THP‐1. The cells were exposed to varying concentrations of ASK for 24 and 48 h. Our results revealed that three tested leukaemia cell lines demonstrated a various proliferation inhibition with ASK treatment in a dose‐dependent pattern. The HL‐60 cells showed more sensitivity to ASK treatment with an IC_50_ of approximately 1.126 μM at 24 h and 0.614 μM at 48 h (Figure 1B). After 24‐h treatment, the cell proliferation was also assessed using the EdU assay. The results showed that ASK significantly inhibited the proliferation of three tested leukaemia cell lines in a concentration‐dependent manner. The results of EdU assay also showed that HL‐60 cells were the most sensitive to ASK (Figure 1C, D).

**FIGURE 2:**
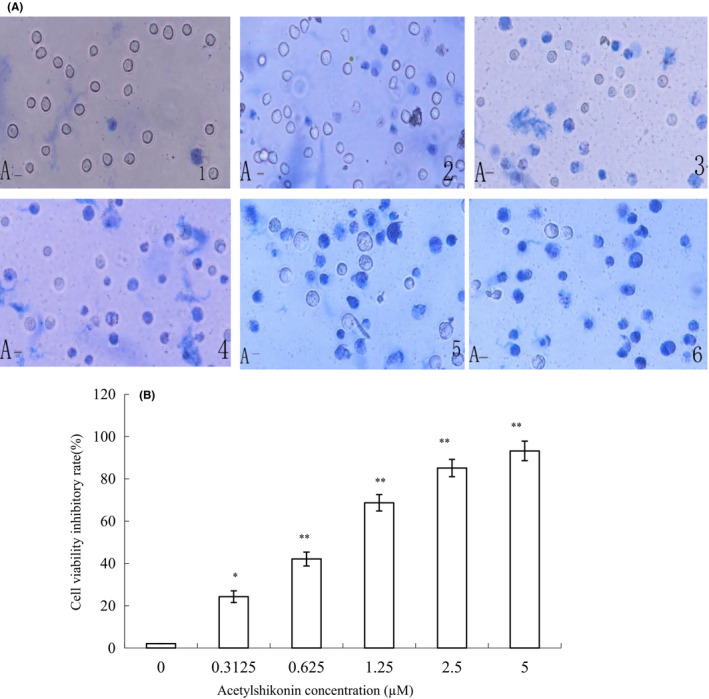
Effects of ASK on HL‐60 cell viability. (A) Morphological evaluation of HL‐60 cells with Trypan blue assay under treatment with ASK (0–5 μM). ×400; Scale bar = 20 µm. (1) Untreated HL‐60 cells; (2–6): HL‐60 cells exposed to 0.3125–5 µM ASK. Non‐survived cells are stained blue. (B) Percentage of cell viability. Mean ± SD. *n* = 3. *p < 0.05, **p < 0.01

We next determined whether HL‐60 cell viability could be affected by ASK. HL‐60 cells were exposed to various concentrations of ASK for 24 h, and cell viability was examined by the trypan blue dye exclusion assay. Our results demonstrated that the untreated HL‐60 cells were viable and were shown to have an obvious cytoplasm (Figure 2A 1), while the majority of 0.3125–5 μM ASK‐treated cells were non‐viable and shown to have a blue cytoplasm (Figure 2A 2‐6). The cell survival results indicated that ASK (0.3125–5 μM) significantly attenuated HL‐60 cell viability in a dose‐dependent fashion (*p *< 0.05; Figure 2B). Interestingly, a low concentration of ASK (0.3125 μM) significantly suppressed the viability of HL‐60 cells. These findings reveal that ASK remarkably inhibits HL‐60 cell viability and induces cell death.

### 3.2 ASK causes S‐phase arrest in HL‐60 cells

**FIGURE 3:**
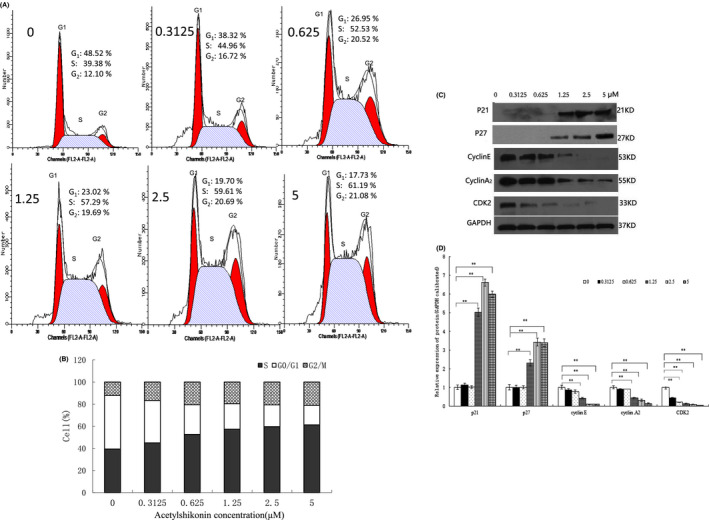
Effects of ASK on HL‐60 cell cycle distribution. (A, B) Cell cycle histograms of HL‐60 cells exposed to ASK (0–5 μM). (C, D) Western blot results showed the expression of p21, p27, cycline E, cycline A2, CDK2 in HL‐60 cells exposed to ASK. The results B and D are presented as Mean ± SD, *n* = 3. *p < 0.05, **p < 0.01

The effect of ASK on the cell cycle distribution in HL‐60 cells was performed with flow cytometry method after PI staining. Treatment with 0.3125–5 μM ASK for 24 h remarkably altered the distributions of cell cycles in HL‐60 cells. As shown in Figure 3A and B, ASK induced the cell cycle arrest of HL‐60 cells at the S phase, along with the decreased percentage of cells at the G1 phase. With increasing ASK doses, the proportion of HL‐60 cells at the S phase gradually increased from 39.38% to 61.19% (*p *< 0.05), while that of HL‐60 cells at the G1 phase reduced from 48.52% to 17.73% (*p *< 0.05). This result suggests that ASK could induce the S‐phase cell cycle arrest of HL‐60 cells.

To explore the mechanisms underlying ASK‐induced cell cycle arrest in HL‐60 cells, the expression levels of cell cycle proteins were detected by immunoblotting. The results showed that, compared with control group, the levels of the cyclin‐dependent kinases (CDKs) cyclin A, cyclin E and CDK2 were decreased in HL‐60 cells, whereas the levels of the CDK inhibitors p21 and p27 were upregulated in ASK treatment groups (Figure 3C, D). Therefore, ASK induced cell cycle arrest in the S‐phase by increasing the protein levels of p21 and p27 and decreasing those of CDK2, cyclin A and cyclin E.

### 3.3 ASK significantly enhanced HL‐60 cell apoptosis

**FIGURE 4:**
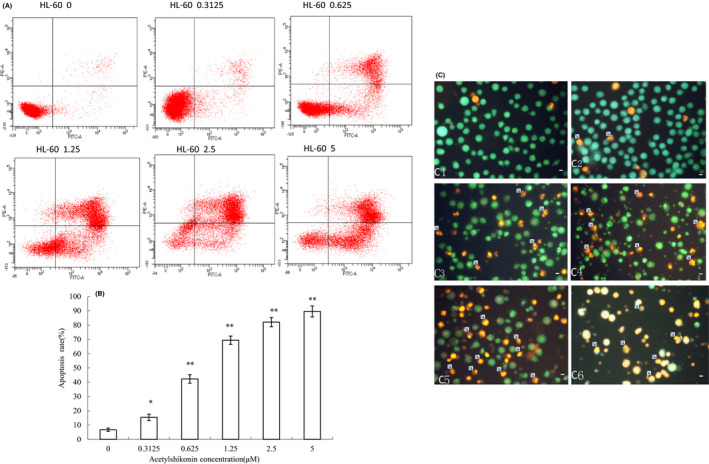
ASK promotes HL‐60 cell apoptosis. The cells were exposed to different concentrations (0–5 μM) of ASK for 24 h. Annexin V/PI apoptosis assay was then carried out. (A) 0, 0.3125, 0.625, 1.25, 2.5 and 5 μM ASK treatment groups. (B) Apoptotic rates of HL‐60 cells exposed to ASK. Mean ± SD. *n* = 3. *p < 0.05, **p < 0.01. (C) Effects of ASK on apoptosis‐related morphological alterations by ethidium bromide (EB)/acridine orange (AO) staining in HL‐60 cells. ×400; Scale bar = 20 µm. (1) Control HL‐60 cells; and (2–6) HL‐60 cells exposed to 0.3125–5 µM ASK. Arrow indicates late apoptosis

Apoptosis is a physiological form of programmed cell death. To confirm the occurrence apoptosis, we analysed apoptotic cells under ASK treatment by flow cytometry afterannex in V‐PI staining. In this experiment, HL‐60 cells were exposed to various concentrations of ASK for 24 h. Our results indicated that the percentage of HL‐60 cell apoptosis gradually increased with increasing ASK concentrations. The percentages of apoptotic HL‐60 cells treated with 0.3125–5 μM ASK were 20.12 ± 2.15%, 43.36 ± 2.87%, 71.91 ± 3.12%, 85.45 ± 3.61% and 89.31 ± 3.06%, respectively, indicating that the apoptotic rate of HL‐60cells treated with ASK was remarkably higher than that (9.82 ± 1.21%) of untreated HL‐60 cells (*p *< 0.05, Figure 4A, B).

EB/AO staining was used to assess the morphology and cell membrane integrity of HL‐60 cells after treatment with ASK. EB can stain the nucleus of cells whose membrane integrity is lost and makes the nucleus appears red, whereas AO permeates the whole cell and makes the nucleus appears green.^26^ HL‐60 cells treated with/without ASK for 24 h were subjected to EB/AO staining, and the amount of apoptotic cells was compared between different treatment lines. As shown in Figure 4C, the nuclear morphology of the untreated cells was normal, suggesting that chromatin was equally distributed in the nuclei. On the contrary, the ASK‐treated cells had apoptotic body formation and nuclear condensation, indicating that cell apoptosis was induced with increasing concentrations of ASK (Figure 4C).

### 3.4 ASK promotes HL‐60 cell apoptosis via mitochondrial pathway

**FIGURE 5:**
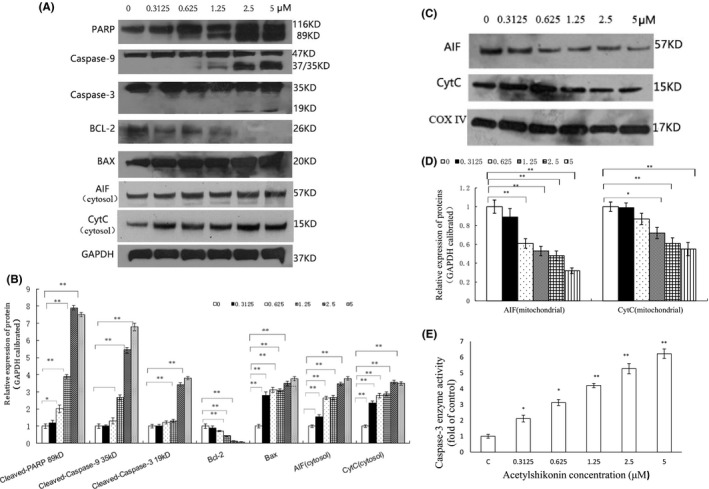
Cleavage and expression of apoptosis‐related proteins in HL‐60 cells exposed to ASK. (A, B) The cleavage of PARP, caspase‐3 and caspase‐9 and protein levels of Bcl‐2, Bax, Cyt C and AIF (in the cytosol). (C, D) Western blot results showed the expression of AIF and Cyt C in the mitochondrial fraction. (E) Caspase‐3 activity under ASK treatment. The results B, D and E are presented as Mean ± SD, *n* = 3. *p < 0.05, **p < 0.01

To elucidate the mechanisms by which ASK promotes cell apoptosis, the cleavage and expression levels of the intrinsic apoptotic pathway‐related proteins were detected after ASK treatment. Our immunoblotting results showed that ASK increased the protein levels of Bax and the cleavage of PARP, caspase‐3 and caspase‐9, which are morphological hallmarks of apoptosis (Figure 5A and B). We also can see that the expression of Bcl‐2 family proteins regulating mitochondrial apoptosis was changed with ASK treatment. The results demonstrated that the expression of Bcl‐2 was downregulated with increasing ASK concentrations, while that of Bax was significantly increased. The ratio of Bax: Bcl‐2 was increased in ASK group in a dose‐dependent fashion (Figure 5A and B). The activity of caspase‐3 in ASK‐treated cells was further verified. Our data also showed that caspase‐3 activities gradually increased with increasing ASK concentrations (Figure 5E).

Apoptosis‐inducing factor (AIF) and cytochrome C (Cyt C) are released from the mitochondria into the cytosol in response to cell apoptosis induced by most chemotherapeutic agents. Therefore, we examined the expression levels of AIF and Cyt C in the cytosolic and mitochondrial fraction by immunoblotting. It was found that the level of Cyt C and AIF were decreased in the mitochondrial fraction (Figure 5C and D), but they were upregulated in the cytosol after ASK treatment (Figure 5A and B). The results indicated that ASK could trigger the release of AIF and Cyt C from the mitochondria into the cytosol. Taking these results into account, we hypothesize that mitochondria play an important role in ASK‐induced HL‐60 cell apoptosis.

### 3.5 ASK promotes autophagy in HL‐60 cells

**FIGURE 6:**
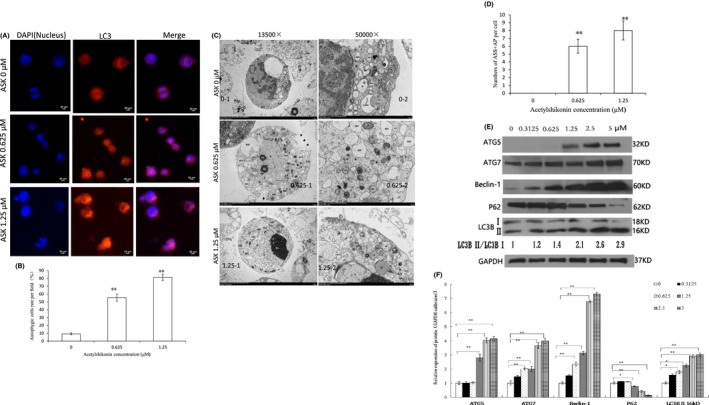
ASK induces autophagy in HL‐60 cells. HL‐60 cells were grown in a 6‐well plate and then exposed to 0.625 and 1.25 µM ASK for 24 h. (A, B) Autophagy marker LC3B was assessed by immunofluorescence after exposure to 0.625 and 1.25 µM ASK for 24 h. Red fluorescence indicates LC3B expression in the cytoplasm, while blue fluorescence represents DAPI in the nucleus. ×400; Scale bar = 20 μm. (C, D) Autophagy structures ASS and AP formation were examined by transmission electron microscopy after treatment with 0.625 and 1.25 µM ASK for 24 h, the left column was detected with magnification of 13 500× and the right column was detected with magnification of 50 000×. Numerous AP and ASS were observed in ASK‐treated cells. ASS, autolysosome; AP, autophagosome; M, mitochondrion; RER, rough endoplasmic reticulum. (E, F) HL‐60 cells were exposed to various concentrations of ASK for 24 h, the conversion from LC3B I to LC3B II and the levels of autophagy‐related proteins were detected by immunoblotting. The result of B, D and F is presented as Mean ± SD, *n* = 3. *p < 0.05, **p < 0.01

Autophagy can promote cell apoptosis or survival under specific circumstances.^27^ To investigate whether ASK can induce cell autophagy, the formation of autophagosomes in HL‐60 cells was detected. Autophagosome formation was confirmed by detecting the localization of the autophagic membrane LC3B in HL‐60 cells by immunofluorescence after treatment with ASK. The LC3B distribution displayed an increased speckled pattern in the cell cytoplasm in the 0.625 and 1.25 μM ASK treatment group (Figure 6A, 0.625 and 1.25 group) compared to the control group (Figure 6A, 0 group). ASK significantly increased the autophagic cell rate, and the autophagic cell rate induced by 1.25 μM ASK was higher than that of 0.625 μM ASK (Figure 6A, B). Autophagosome formation after ASK exposure was also verified by TEM. After treatment with 0.625 and 1.25 μM ASK for 24 h, the formation of double‐membrane bound autophagic vesicles was detected in ASK‐treated cells (Figure 6C, 0.625–1,2 and 1.25–1,2), but not in control cells (Figure 6C, 0–1,2). Organelles were visible within double‐membrane autophagic vesicles at high magnifications (Figure 6C, 0.625–2 and 1.25–2). In addition, an increased number of autophagy structures ASS (autolysosome) and AP (autophagosome) were observed in the ASK treatment groups (Figures 6A, 0.625–1,2 and 1.25–1,2) compared to the control group (Figure 6C, 0–1,2). The number of ASS and AP after 1.25 μM treatment was higher than that of 0.625 μM treatment (Figure 6C, D). These findings indicated that autophagy is induced in HL‐60 cells exposed to ASK treatment.

To identify further evidence supporting the formation of autophagosomes and modulation of macroautophagic flux in HL‐60 cells following ASK treatment, the turnover from LC3B I to LC3B II was detected. Western blotting results revealed that ASK‐treated HL‐60 cells exhibited a higher ratio of LC3B II over LC3B I than the control cells (Figure 6E). We also observed that the expression of other autophagy‐related genes (e.g. Beclin‐1, ATG5 and ATG7) was upregulated after exposure to ASK in a dose‐dependent fashion (Figure 6E, F). However, the expression of P62 was decreased in a dose‐dependent fashion (Figure 6E, F). These findings demonstrate that ASK treatment induces the autophagic process of HL‐60 cells.

### 3.6 ASK promotes autophagy by upregulating LKB1/AMPK and downregulating PI3K/Akt/mTOR pathways in HL‐60 cells

**FIGURE 7:**
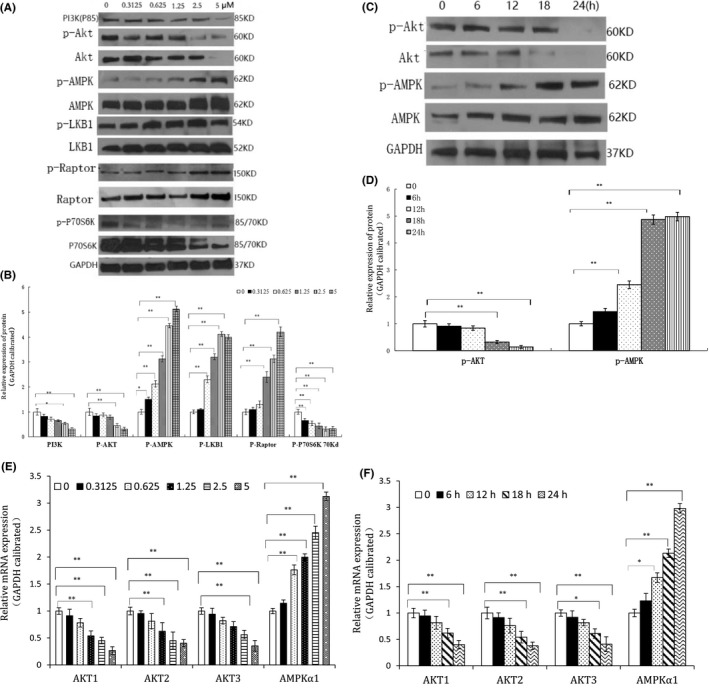
HL‐60 cell autophagy induced by ASK is dependent on a role of LKB1/AMPK and PI3K/Akt/mTOR signalling pathways. (A, B) P‐AMPK/AMPK, p‐Akt/Akt, PI3K (P85), p‐p70S6K/p70S6K, p‐Raptor/Raptor and p‐LKB1/LKB1 were assessed by immunoblotting after HL‐60 cells were exposed to various concentrations of ASK for 24 h. (C, D) P‐AMPK/AMPK, p‐Akt/Akt were assessed by immunoblotting after HL‐60 cells were exposed to 1.25 μM ASK for different times. (E) Akt (including Akt1, Akt2 and Akt3) and MAPKα1 mRNA expression were analysed by qRT‐PCR after HL‐60 cells were exposed to various concentrations of ASK for 24 h. (F) Akt and MAPKα1 mRNA expression were analysed by qRT‐PCR after HL‐60 cells were exposed to 1.25 μM ASK for different times. The results B, D, E and F are presented as Mean ± SD, *n* = 3. *p < 0.05, **p < 0.01

To elucidate the underlying mechanisms of ASK‐regulated autophagy, LKB1/AMPK and PI3K/Akt/mTOR signalling pathways were assessed in HL‐60 cells treated with ASK. Figure 7A–D showed that the PI3K‐Akt signalling was suppressed after ASK treatment, as shown by probing with antibodies of p‐Akt and PI3K (p85). However, the LKB1/AMPK signalling was activated by ASK, as revealed by the increased expression of p‐LKB1 and p‐AMPK. The expression of p‐Akt was decreased after treatment with ASK in a time‐ and dose‐dependent fashion, while p‐AMPK and p‐LKB1 protein expression was increased (Figure 7A–D). Analysis of quantitative real‐time RT‐PCR results showed that gene expression of Akt (including Akt1, Akt2 and Akt3) was decreased and MAPKα1 was increased under the action of ASK in a dose‐ and time‐dependent manner, which is consistent with the western blot results (Figure 7E, F). This indicated that the changes of Akt and AMPK total protein expression induced by ASK were caused at the gene expression level. The levels of phospho‐p70S6K, a protein downstream of mTOR, decreased in a similar manner to the p‐Akt protein (Figure 7A, B). AMPK can phosphorylate Raptor directly, and this phosphorylation is required for the suppression of raptor‐containing mTOR.^28^ It was observed that p‐Raptor was upregulated after treatment with ASK (Figure 7A, B), which was consistent with the activation of p‐AMPK induced by ASK. From the above data, we can see that both the LKB1/AMPK and PI3K/Akt pathways are involved in the ASK‐induced autophagy.

**FIGURE 8:**
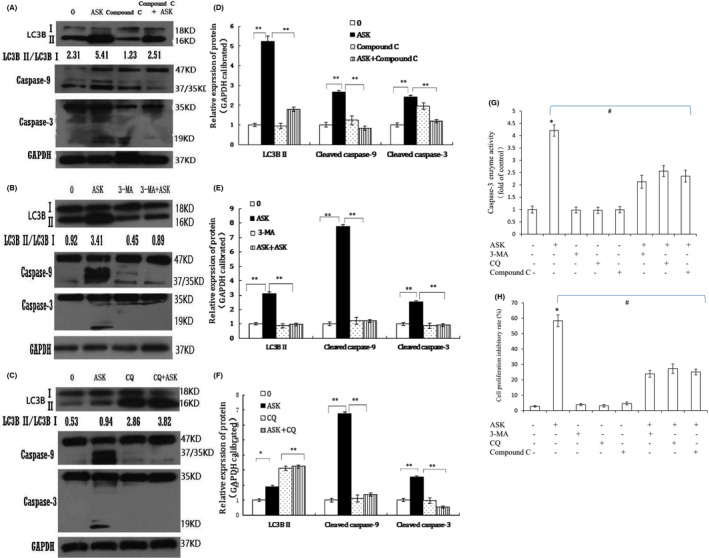
HL‐60 cells were exposed to 1.25 μM ASK in the combination of 5 μM compound C (A, D), 2 mM 3‐MA (B, E) and 10 μM CQ (C, F) for 24 h, then the conversion from LC3B I to LC3B II and the cleavage of PARP, caspase‐3 and caspase‐9 were analysed by immunoblotting. The results D, E and F are presented as Mean ± SD, *n* = 3. *p < 0.05, **p < 0.01. Caspase‐3 activity was determined by commercial kit (G), and cell proliferation was analysed by XTT assay (H). Mean ± SD. *n* = 3. *p < 0.05 vs. normal group; #*p *< 0.05 vs. ASK‐exposed cells

We further proved that ASK can induce autophagy through activating LKB1/AMPK/ signalling by application of an AMPK inhibitor compound C combined with ASK in HL‐60 cells. Compound C, a reversible selective inhibitor of AMPK, was used to prove that the increase of AMPK phosphorylation was involved in the inhibition of mTOR during autophagy.^29^ The cells were pre‐exposed to 5 µM compound C (AMPK inhibitor) for 2 h, followed by 1.25 µM ASK for 24 h. Western blotting results showed that ASK‐induced the conversion of LC3B from type I to type II was reduced in compound C group combined with ASK compared with ASK alone (Figure 8A, D). Based on the fact that compound C inhibited the function of AMPK, the conversion of LC3B‐I to LC3B‐II induced by ASK is reduced to the original level when combined with compound C. Based on the above results, we can see that that ASK‐induced autophagy requires activation of AMPK‐LKB1 pathway and inhibition of PI3K/Akt pathway.

### 3.7 Acetylshikonin‐induced autophagy enhances HL‐60 cell apoptotic cell death

The above data indicated that ASK promotes both apoptosis and autophagy in HL‐60 cells. To elucidate the relationships between apoptosis and autophagy, the cells were pretreated with the inhibitor of AMPK (compound C) (5 µM), an autophagy inhibitor 3‐MA (2 mM) and another autophagy inhibitor CQ (10 µM) for 2 h followed by 1.25 µM ASK for 24 h, and we detected the turnover from LC3B I to LC3B II and the cleavage of caspase‐3 and caspase‐9 compared with that in cells treated with ASK alone. It has been well accepted that 3‐MA is a widely used inhibitor of autophagy via its inhibitory effect on class III PI3K.^30^ CQ inhibits autophagy by neutralizing lysosomal pH and inhibiting the fusion of autophagosomes and lysosomes.^31^ Therefore, CQ is also often used as another inhibitor of autophagy. Western blotting results showed that ASK‐induced the conversion of LC3B from type I to type II was reduced in 3‐MA and compound C groups combined with ASK compared with ASK alone (Figure 8A, D and B, E), while combined treatment with CQ and ASK, ASK‐induced the turnover from LC3B I to LC3B II was upregulated compared with ASK alone (Figure 8C, F). Because 3‐MA inhibit autophagy by inhibitory effect on class III PI3K, the conversion from LC3B I to LC3B II induced by ASK is reduced to the original level when ASK combined with 3‐MA. Since CQ inhibits the binding of autophagosomes to lysosomes, the higher ratio of LC3B II over LC3B I was detected when combination with CQ compared with ASK alone. We can also see that the cleavage of caspase‐3 and caspase‐9 was decreased when 3‐mA, CQ and compound C were combined with ASK compared with ASK alone (Figure 8A–F). Caspase‐3 enzyme activities were decreased with ASK treatment in the presence of 3‐MA, CQ and compound C in comparison with the group treated with ASK alone (Figure 8G). From these data, we can conclude 3‐MA, CQ and compound C inhibits apoptosis, indicating that the HL‐60 cells apoptosis induced by ASK should be enhanced by autophagy. To determine whether ASK induced cell death through autophagy, 3‐MA, CQ and compound C were used. The findings demonstrated that the anti‐proliferation effects of ASK on HL‐60 cell were decreased under cotreatment with ASK and 3‐MA, CQ and compound C in comparison with the group treated with ASK alone (Figure 8H). From the above data, we preliminarily concluded that the autophagy induced by ASK can enhance the apoptotic cell death of HL‐60 cells.

## 4 DISCUSSION

Acetylshikonin exhibits various biological activities and therapeutic potentials. Several studies have reported that ASK can induce apoptosis in different cancer cells and exhibit antitumour activities against oral squamous cells, melanoma cells and colorectal cells.^19^, ^20^, ^21^, ^22^ Our previous study showed that ASK can inhibit the proliferation of human chronic myelogenous leukaemia K562 cells and induce apoptosis by blocking NF‐κB signalling and depleting Bcr‐Abl.^32^ Increasing attention has been paid to the mechanism involved in ASK‐induced autophagy. Wang and colleagues^33^ reported that ASK could suppress the growth of cisplatin‐resistant oral squamous cell carcinoma via apoptosis and autophagy. In this work, we described that ASK triggered both cell autophagy and apoptosis in the acute myelogenous leukaemia FAB‐M2 cell line HL‐60 through the key LKB1‐AMPK and PI3K/Akt‐regulated mTOR signalling pathways, which could better understand the relationship between autophagy and apoptosis during ASK‐induced cell death.

It was found that ASK suppressed the proliferation of HL‐60 cells in a time‐ and dose‐dependent fashion (Figure 1). The trypan blue exclusion results demonstrated that low concentrations of ASK remarkably affected HL‐60 cell viability (Figure 2).

The suppressive effects of ASK on HL‐60 cell growth may be through apoptotic induction and cell cycle arrest. Our finding indicated that ASK promoted HL‐60 cell cycle arrest in the S‐phase (Figure 3). It has been reported that shikonin suppresses tumour cell proliferation by promoting cell cycle arrest as Topo I inhibitor,^34^ such as our previous study.^32^ Zhang and colleagues also demonstrated that shikonin as Topo I inhibitor induced the cell cycle arrest at the S phase.^35^ So we speculated that ASK should be employed as a Topo I inhibitor in HL‐60 cells, thus blocking DNA synthesis at the S phase. Moreover, we found ASK might modulate PI3K/Akt and AMPK pathway resulting in autophagy‐dependent apoptosis. Our results may benefit to use ASK to overcome topoisomerase inhibitor‐resistant cancers. The progression of cell cycles is regulated by the coordinated activity of CDKs and regulatory cyclins.^36^ The G1/S phase transition is mainly dependent on cyclin A/CDK2 and cyclin E/CDK2 kinase complex activities, which can be blocked by p21 and p27.^37^, ^38^, ^39^, ^40^ In the present study, the downregulated expression of cyclin A, cyclin E and CDK2 and the upregulated expression of p21 and p27 were consistent with the increased S‐phase arrest (Figure 3).

Apoptosis induction should be another potential mechanisms underlying the antiproliferative effects of ASK on HL‐60 cells. Our results of apoptosis rates and typical morphological changes of apoptotic cells indicated that 0.3125–5 μM ASK significantly induced HL‐60 cell apoptosis (Figures 4, 5).

Moreover, our findings also demonstrated that ASK induces autophagy in HL‐60 cells. The turnover from LC3B I to LC3B II is usually investigated as the hallmark of the autophagic membrane that monitors autophagosome formation at the early stages.^41^ ASK enhanced the conversion from LC3B I to LC3B II and protein levels of Atg5, Atg7 and Beclin‐1 while decreasing P62 expression (Figure 6E and F) in HL‐60 cells. Autophagosome formation was also confirmed by detecting the localization of LC3B using immunofluorescence method and by detecting autophagy structures ASS and AP formation using TEM method in HL‐60 cells after treatment with ASK (Figure 6A–D). Therefore, HL‐60 cell death induced by ASK was triggered by both apoptotic and autophagic machinery. Notably, ASK was shown to trigger both apoptosis and autophagy in human oral cancer cells.^33^ Thus, our study is consistent with previous findings.

Beclin‐1 and Bcl‐2 family pathways have been reported to involve in the regulation of autophagy in cancer cells.^42^ The capability of Beclin‐1 to promote autophagy or apoptosis is negatively regulated by Bcl‐2 via BH3 domain binding.^43^ Our results showed that the expression of Beclin‐1 protein was upregulated by ASK treatment. In addition, Bcl‐2 expression was suppressed by ASK, while Bax expression was increased to some extent. These results indicated that ASK could promote HL‐60 autophagy or apoptosis through enhancing the Beclin‐1 release from its binding with Bcl‐2 via the suppression of Bcl‐2. Furthermore, we also found that treatment with ASK could exert apoptotic effects on HL‐60 cells via PARP cleavage and caspase‐3 activation. Bcl‐2 is also recognized as one of the anti‐apoptosis family proteins.^44^ Bax/Bcl‐2 can regulate the release of Cyt C from mitochondria into the cytosol, and subsequently activates the caspase cascades (e.g. caspase‐3 and caspase‐9), thus leading to cell apoptosis.^45^ Caspase‐9 is activated by Cyt C in the intrinsic pathway^46^ and has the unique ability to activate effector caspases (e.g. caspase‐3, caspase‐6 and caspase‐7).^47^ PARP is then cleaved by the activated caspase‐3, which in turn leads to cell apoptosis.^48^ Our findings showed that ASK remarkably upregulated the cleavage of PARP, caspase‐3 and caspase‐9 in a dose‐dependent fashion, together with the increased expression of AIF and Cyt C in the cytosol (Figure 5). These findings are in good agreement with those of previous studies,^46^, ^49^ indicating that ASK‐treated HL‐60 cells underwent apoptosis through the mitochondria‐mediated intrinsic pathway. Thus, it can be inferred that ASK induces autophagic and apoptotic cell death by inhibiting Bcl‐2 interaction with Beclin‐1 in HL‐60 cells.

Akt and AMPK are essential for the regulation of cell apoptosis and autophagy.^50^ It is generally agreed that the process of autophagy is mediated through the PI3K/Akt/mTOR signalling pathway. Inhibition of Akt signalling is crucial for the development of therapeutic agents against tumour cells.^51^ Zhao and co‐workers^52^ found that MK‐2206 (an Akt inhibitor) could induce autophagy in human nasopharyngeal cancer cells. Currently, AMPK can act as a negative regulator of mTOR to induce autophagy by directly modulating Beclin‐1 and ULK1.^53^ AMPK regulate the ULK1 complex by downregulating the raptor activation, a direct substrate of AMPK and mTOR complex 1.^53^ The phosphorylation of raptor is required for the suppression of raptor‐containing mTOR. In our results, ASK decreased the p‐Akt expression and induced the expression of p‐AMPK, p‐raptor and Beclin‐1 (Figures 6E and 7). In addition, we observed that compound C (an AMPK inhibitor) potently inhibited the conversion from LC3B I to LC3B II induced by ASK (Figure 8A, C). Therefore, we confirmed that ASK promoted autophagic cell death through activation of the LKB1/AMPK pathway and suppression of PI3K/Akt/mTOR signalling in HL‐60 cells. The results of Liu and colleagues^54^ showed that AA005 (an AMPK activator) could induce autophagy in colon cancer cells via suppression of mTORC1, which were consistent with our results.

As suggested above, we can see that ASK could induce apoptosis and autophagy by increasing the cleavage of PARP, caspase‐3 and caspase‐9 and the turnover from LC3B I to LC3B II in HL‐60 cells via activation of LKB1/AMPK pathways and suppression of PI3K/Akt/mTOR signalling pathway. Our data showed that apoptosis and autophagy pathways are both activated and enhanced by ASK, which is consistent with previous reports.^33^, ^55^, ^56^ These results led us to study the potential relationship between autophagy and apoptosis processes.

Apoptosis is associated with the mitochondria‐integrated cell death signals and autophagy activation.^57^ For instance, mitochondrial permeability transition can cause interconnection between apoptotic and autophagic cell death, and resveratrol‐induced apoptosis could be regulated by caspase‐3 activity and Cyt C level.^58^ Our results showed that ASK significantly elevated caspase‐3 activities in a dose‐dependent fashion (Figure 5E), and ASK induced HL‐60 apoptotic cell death regulated by the mitochondrial intrinsic apoptotic pathway. We also found that the cleavage of caspase‐3 and caspase‐9 and the activity of caspase‐3 were decreased when 3‐mA, CQ and compound C were combined with ASK treatment compared with ASK treatment alone. This indicated that ASK‐induced apoptosis is reduced after the inhibition of autophagy by pharmaceutical inhibitors of autophagy. Our findings also revealed that the inhibition of HL‐60 cell proliferation decreased after ASK treatment combined with 3‐MA, CQ and compound C. From these experimental data, we suggest that autophagy induced by ASK can serve as important process by which to promote HL‐60 apoptotic cell death, as suggested by others.^55^, ^59^

## 5 CONCLUSION

In summary, our findings demonstrated that ASK can inhibit the proliferation, induce the apoptosis and autophagy of human acute myelogenous leukaemia (AML) HL‐60 cells. ASK effectively promoted cell cycle arrest at the S phase and suppressed cell proliferation through apoptosis and autophagy in vitro. Moreover, activation of LKB1/AMPK and suppression of PI3K/Akt/mTOR signalling pathway played a crucial role in the treatment with ASK. The mechanism underlying ASK‐induced HL‐60 cell death may occur through autophagy, and ASK‐induced autophagy may be a necessary process for apoptosis. However, the experiments were done only in cellular level. Further investigation is needed to completely elucidate this observation in vivo. Our findings may shed light on the underlying mechanisms of ASK and propose its therapeutic potentials. Exploring the key pathways underlying the inhibitory effects of ASK on HL‐60 cells give an idea as a candidate in developing novel human leukaemia therapeutic alternatives with less side effects.

## CONFLICT OF INTEREST

The authors declare that they have no competing interests.

## AUTHOR CONTRIBUTIONS

**wu mengdi:** Data curation (equal); Formal analysis (equal); Investigation (equal); Methodology (equal); Writing – original draft (equal). **zhang yuanying:** Data curation (equal); Investigation (equal). **yi shuying:** Data curation (equal); Investigation (equal). **sun beibei:** Data curation (equal). **lan jing:** Data curation (equal); Investigation (equal). **hanming jiang:** Conceptualization (equal); Supervision (equal); Writing – review & editing (equal). **hao gangping:** Conceptualization (equal); Data curation (equal); Formal analysis (equal); Funding acquisition (equal); Investigation (equal); Methodology (equal); Project administration (equal); Resources (equal); Supervision (equal); Visualization (equal); Writing – original draft (equal); Writing – review & editing (equal).
